# Heat-Not-Burn Tobacco Products Are Getting Hot in Italy

**DOI:** 10.2188/jea.JE20180040

**Published:** 2018-05-05

**Authors:** Xiaoqiu Liu, Alessandra Lugo, Lorenzo Spizzichino, Takahiro Tabuchi, Giuseppe Gorini, Silvano Gallus

**Affiliations:** 1 Department of Environmental Health Sciences, IRCCS - Istituto di Ricerche Farmacologiche “Mario Negri”, Milan, Italy; 2 Prevention Department, Ministry of Health, Rome, Italy; 3 Cancer Control Center, Osaka International Cancer Institute, Osaka, Japan; 4 Oncological network, prevention and research institute (ISPRO), Florence, Italy

**Keywords:** heat-not-burn tobacco products, IQOS, legal sales trends, Italy

Dear Editor,

We read with great interest that the *Journal of Epidemiology* has recently decided not to consider for publication manuscripts on research carried out with funding from the tobacco industry.^1^ We strongly support such a decision. Until now, we have seen a lot of studies conducted by the tobacco industry, which are likely to be biased due to conflicts of interest. New tobacco products are not exempt from this phenomenon.^2^ Indeed, most of the knowledge on heat-not-burn tobacco products (HNB) comes from the tobacco industry.^3^^,^^4^

HNBs are hybrids between electronic and conventional cigarettes: on one hand, they are equipped with a device that heats the product, without reaching combustion, to generate aerosol (ie, a sort of “cold smoke”); on the other hand, the product used is not a liquid containing nicotine, but “real” tobacco.^4^^,^^5^ IQOS is the brand name of such a product by Philip Morris International (PMI). IQOS has pioneered the HNB market since December 2014, after having been launched in test markets in Milan (Italy) and Nagoya (Japan). To date, it is in commerce in 30 countries, including 22 from the WHO European region.^4^^,^^6^ In Italy, IQOS expanded the market to the whole country since December 2015, and, until December 2017, it was the only available HNB. We provide, hereby, independent data on sales of HNBs in Italy.

**Figure 1.**
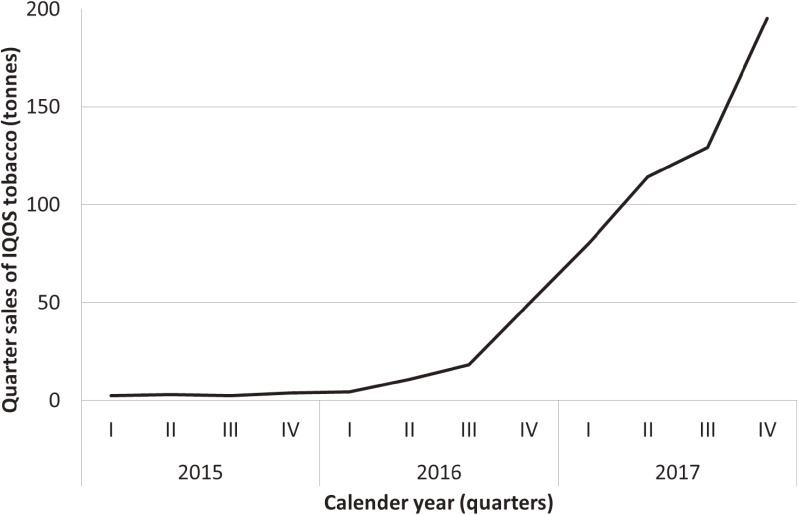
Quarter sales of IQOS tobacco (tonnes), Italy, 2015–2017

Legal sales data of HNBs, obtained by the Italian Ministry of Finance, showed that the annual sale of IQOS remained negligible in 2015 (11 tonnes per year). Subsequently, it grew to 83 tonnes in 2016, up to 519 tonnes in 2017 (Figure 1). Correspondingly, the market share of IQOS in the whole tobacco market increased from 0.01% in 2015 to 0.11% in 2016 and up to 0.67% in 2017 and is now approaching the market share of cigars.

These data reveal a quick exponential increase in IQOS sales in Italy over the last 3 years. This increase parallels the total IQOS online search-volume across Italy, according to Google Trends (https://trends.google.com/trends/). IQOS online search-volume was in fact boosted by more than 10 times in a single year (2016) and continued to increase in 2017.

These trends may be of concern, since we have previously shown that nearly half of Italian IQOS users (45%) and over half of the people interested in IQOS (51%) are never smokers.^4^ Therefore, such a product may represent, at least in Italy, a gateway for nicotine addiction among never smokers rather than a harm reduction substitution for current smokers.^4^ Moreover, the few independent toxicological studies have consistently found that HNBs release relatively high nicotine levels (similar to those released by conventional cigarettes)^5^^,^^7^ and non-negligible amounts of harmful substances, including various carcinogens.^5^^,^^8^ Nevertheless, due to the alleged belief in HNB harm reduction in Italy, these new products are exempted from the fiscal regimes of tobacco products. In fact, HNBs enjoy the same tax reduction as electronic cigarettes, which is half that of conventional cigarettes.^9^ Moreover, the enforcement of various tobacco control regulations is only minimally adopted for HNBs in Italy. First of all, health warnings are required to cover only 30% of the HNB packaging (instead of 65% for conventional cigarettes), without pictorial images.^10^ Second, comprehensive smoke-free regulations prohibiting smoking in all public places and workplaces do not apply to HNBs.^9^^,^^10^ Finally, advertising and promotions are not banned for these new products. This is evident by the presence in several strategic Italian cities of the “IQOS embassy” and “IQOS boutique”, which are fancy concept stores where IQOS is promoted as a status symbol and people can try it for free. Therefore, the most recognized tobacco control policies (ie, price/tax increase, smoking bans, advertising bans, and health warnings) have been compromised for HNBs in Italy.

In conclusion, although the share of IQOS in the whole Italian tobacco market is still limited, given the exponential increase in sales observed over the last 3 years and the fiscal and regulatory benefits IQOS has,^9^^,^^10^ we expect a further expansion of IQOS in the Italian tobacco market, similar to that recently observed in Japan.^6^
